# Role of listeriolysin O and phospholipases C in *L. monocytogenes* intercellular protrusion dynamics, resolution, and autophagy avoidance

**DOI:** 10.1128/mbio.01183-25

**Published:** 2025-08-18

**Authors:** Madalyn Fields, Abigail Spencer, Sara J. Mason, Christopher C. Phelps, Xiaoli Zhang, Amal Amer, Stephanie Seveau

**Affiliations:** 1 Department of Microbial Infection and Immunity, The Ohio State University Medical College, Columbus, Ohio, USA; 2 Center for Microbe and Immunity Research, Abigail Wexner Research Institute at Nationwide Children’s Hospital, Columbus, Ohio, USA; 3 Biostatistics Core, College of Nursing, University of South Florida 7831, Tampa, Florida, USA; University of Illinois Chicago, Chicago, Illinois, USA

**Keywords:** *Listeria monocytogenes*, cell-to-cell spreading, listeriolysin O, phospholipase C, *Listeria* protrusion, bacterial intercellular spreading

## Abstract

Cell-to-cell spread is a major mechanism used by the bacterial pathogen *Listeria monocytogenes* to disseminate within its host. In this mechanism, bacteria are directly transferred from the cytosol of an infected donor cell to a recipient cell via formation of an intercellular protrusion. The intercellular protrusion resolves into a vacuole that is disrupted by *L. monocytogenes* to reach the cytosol of the recipient cell, where it divides and starts new cell-to-cell spread cycles. Here, we studied the spatiotemporal dynamics of the donor and recipient host cell plasma membranes during intercellular protrusion formation, resolution, and disruption, as well as the role of the bacterial pore-forming toxin listeriolysin O (LLO) and two phospholipases C in these dynamics. Leveraging three-dimensional live-cell microscopy, we co-cultured and imaged infected epithelial cells expressing different fluorescent plasma membrane markers to distinguish donor from recipient cell membranes. We characterized, for the first time, the dynamics of the canonical cell-to-cell spread pathway in which protrusions resolve into double-membrane vacuoles, followed by the successive disruption of donor and recipient membranes. We found that LLO controls protrusion resolution into a double-membrane vacuole and disruption of donor and recipient membranes, whereas the phospholipases C (PLCs) control donor membrane disruption and prevent bacterial entrapment into autophagosomes. Furthermore, we identified a second, more efficient “non-canonical” cell-to-cell spread pathway strictly requiring LLO/PLCs cooperation, which resolves into a single-membrane vacuole that is rapidly disrupted. Altogether, this study provides the first detailed analysis of membrane dynamics within intercellular protrusions and vacuoles, establishing an advanced model for *L. monocytogenes* cell-to-cell spread.

**IMPORTANCE:**

Intracellular bacterial pathogens, including *L. monocytogenes*, spread from cell to cell to effectively disseminate within host tissues and cause systemic infections. We developed a fluorescence microscopy-based method to visualize and distinguish in real time the dynamics of donor and recipient host cell plasma membranes during the different stages of the cell-to-cell spread process: (i) formation of the intercellular protrusion-containing *L. monocytogenes*, (ii) protrusion resolution into a vacuole, and (iii) disruption of the vacuolar membranes until the bacterium is released into the cytosol of the recipient cell. We measured the kinetics of the two identified—canonical and non-canonical—cell-to-cell spread pathways of *L. monocytogenes* in epithelial cells. This work also revealed that the virulence factors listeriolysin O and phospholipases C differentially control donor and recipient membrane remodeling and bacterial capture into autophagosomes, providing an advanced model for *L. monocytogenes* cell-to-cell spread.

## INTRODUCTION

Intracellular bacterial pathogens, including *Listeria monocytogenes* (*Lm*) and *Shigella flexneri*, *Rickettsia*, and *Burkholderia* spp., directly spread from cell to cell to establish systemic infections and cause severe diseases (1–3). Intercellular spreading allows for productive bacterial dissemination within host tissues while avoiding bacterial exposure to numerous extracellular antimicrobial agents. A key aspect of cell-to-cell spread is the manipulation of the host cell cytoskeleton and plasma membrane structures by the pathogen to form a double-membrane intercellular protrusion that invaginates into an adjacent cell, resolving into a vacuole, from which the pathogen escapes to multiply in the cytosol of the newly infected recipient cell (1, 4, 5).

*L. monocytogenes* is the causative agent of listeriosis, a foodborne illness responsible for severe systemic infections in elderly, pregnant, and immunocompromised individuals (6, 7). Central to pathogenesis, *L. monocytogenes* uses multiple virulence factors that exploit the host cell machineries to promote its internalization, cytosolic replication, and intercellular spread (8, 9). Both intracellular replication and cell-to-cell spread are essential for *L. monocytogenes* pathogenesis. Indeed, in the murine model, *L. monocytogenes* mutants unable to grow intracellularly are avirulent, and the 50% lethal dose (LD50) of *L. monocytogenes* mutants defective for cell-to-cell spread is 1,000-fold higher than control wild-type (WT) bacteria (10–13). Virulence factors that promote *L. monocytogenes* cell-to-cell spread are the surface protein ActA (encoded by the gene *actA*) and several secreted proteins: internalin C (InlC, encoded by *inlC*), two phospholipases C (phosphatidylinositol-specific phospholipase C [PI-PLC] and the broad-range phosphatidylcholine-preferring phospholipase C [PC-PLC] encoded respectively by *plcA* and *plcB*), and the pore-forming toxin listeriolysin O (LLO, encoded by *hly*) (2, 3, 14, 15). Each of these bacterial virulence factors mediates specific stages of the cell-to-cell spread process. ActA induces the formation of an actin comet tail at the bacterial pole, generating propulsive forces that ultimately push the bacterium against the plasma membranes of donor and recipient cells to form a double-membrane intercellular protrusion (16–19). Structural stability of the protrusion requires the anchorage of the actin comet tail to the donor cell plasma membrane by actin-binding proteins of the ezrin, radixin, and moesin family (20). Furthermore, InlC stimulates exocytosis and interacts with the host protein, Tuba, to decrease surface tension, facilitating elongation of the donor cell plasma membrane (21–23). Elongation of the recipient cell plasma membrane within protrusions has been proposed to involve mDia-dependent assembly of the actin cytoskeleton and the flattening of caveolae (24–26). Based on electron microscopy analyses, it has been proposed that the intercellular protrusion resolves into a double-membrane vacuole (DMV) (27), the disruption of which is mediated by three secreted virulence factors: PI-PLC, PC-PLC, and LLO (27–30). Based on analyses of *L. monocytogenes* LLO-deficient and PLC (PI-PLC and PC-PLC)-deficient strains, the current model proposes that PLCs are responsible for the disruption of the donor cell membrane, while LLO controls removal of the recipient cell membrane (30).

While significant progress has been made in identifying bacterial and host factors that contribute to *L. monocytogenes* protrusion formation and elongation, little is known about the mechanism of protrusion resolution, and no studies have distinguished the dynamics of the donor from the recipient host cell plasma membranes. Therefore, the roles of LLO and PLCs in the remodeling of donor and recipient membranes at each cell-to-cell spread stage have not been directly established. In the present study, we investigated by three-dimensional (3D) fluorescence microscopy the dynamics of donor and recipient host cell plasma membranes labeled with distinct fluorochromes during *L. monocytogenes* cell-to-cell spread in human HeLa epithelial cells. Cell-to-cell spread events were characterized across wild-type and isogenic *L. monocytogenes* deletion mutants to dissect the roles of LLO and PLCs. Our findings shed new light on *L. monocytogenes* cell-to-cell spread mechanisms, in addition to confirming previously proposed mechanisms. We identified two pathways for protrusion resolution: (i) the canonical pathway in which the protrusion resolves into a DMV and (ii) a significantly faster non-canonical pathway in which the protrusion resolves into a single-membrane vacuole (SMV). The non-canonical pathway strictly requires LLO and PLCs. Importantly, LLO is critical for efficient closure of the protrusion into a DMV and disruption of both donor and recipient membranes, while PLCs are important for the disruption of the donor membrane and prevention of bacterial entrapment into an LC3-positive autophagy compartment during cell-to-cell spread.

## RESULTS

### Role of LLO and PLCs in formation of infectious foci

**Fig 1:**
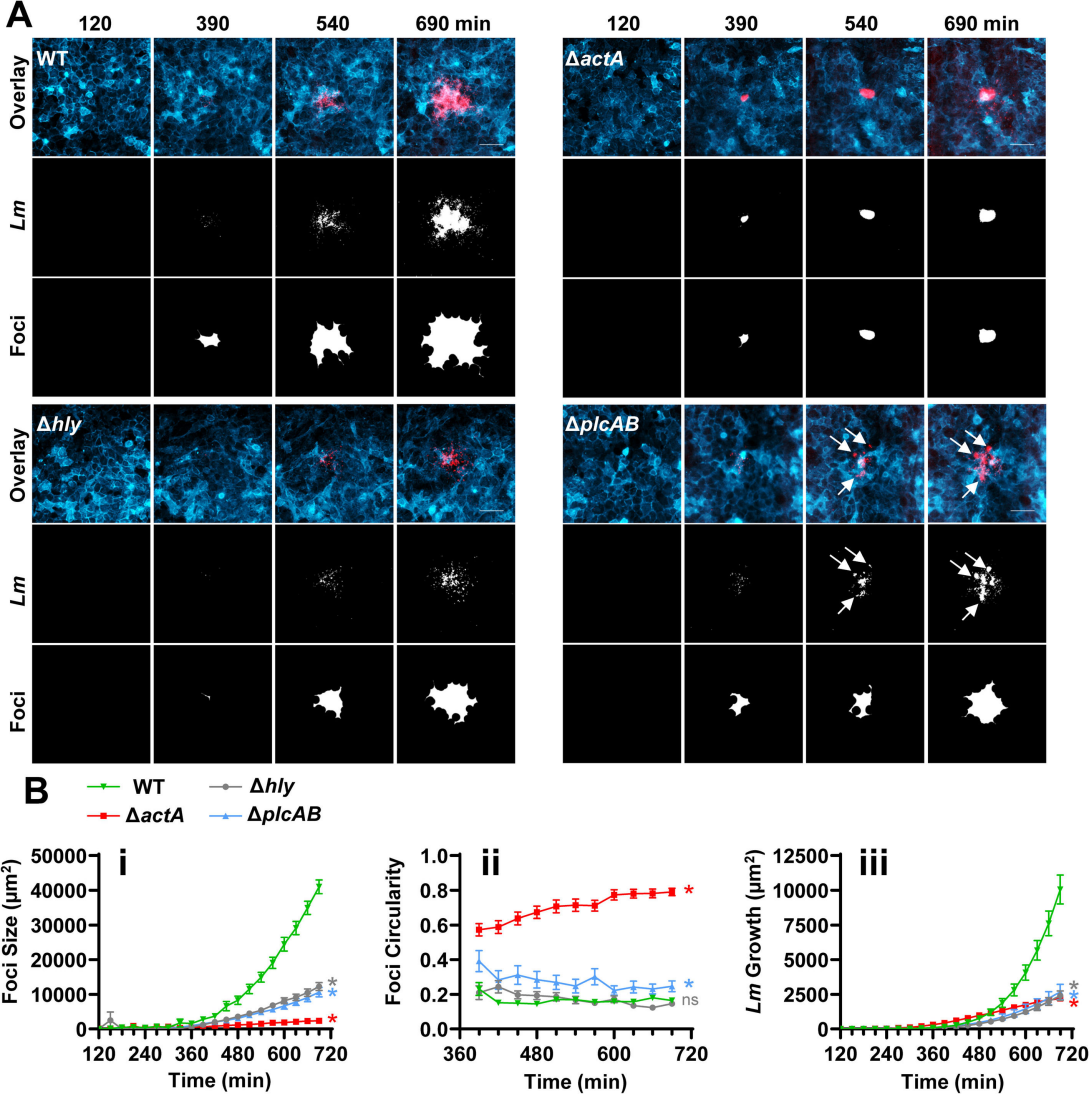
LLO and PLCs control formation of *L. monocytogenes* infectious foci. Plasma membrane-labeled HeLa cells (Lck-mTurquoise2, blue) were infected with WT, ∆*actA*, ∆*hly*, or ∆*plcAB L. monocytogenes* that express RFP upon access to the cytosol (red). (**A**) Micrographs extracted from representative movies (Movie S1) show the progression of infectious foci at four different time points (overlay). Corresponding binary images of fluorescent intracellular *L. monocytogenes* (Bi*Lm*, white) and binary images that encompass the focus regions (Bi*Lm*2, white) are presented below the micrographs. Scale bars are 100 µm. Arrows point out areas of compacted bacterial growth. (**B**i) Average *L. monocytogenes* focus size over time. (Bii) Average focus circularity (perfect circle = 1) over time. (Biii) Average *L. monocytogenes* growth over time. Error bars indicate the standard error of the mean. *N* = 3 independent experiments with 7–10 foci analyzed per strain and independent experiment. Data compared WT to mutant strains by a linear mixed-effects model (*P < 0.05). ns, not significant. (Bi) ∆*actA P* = 0.0001, ∆*hly P* ≤ 0.0001, ∆*plcAB P* = 0.0062; (Bii) ∆*actA P* ≤ 0.0001, ∆*hly P* = 0.5872, ∆*plcAB P* ≤ 0.0001; (Biii) ∆*actA P* ≤ 0.0001, ∆*hly P* = 0.0002, ∆*plcAB P* = 0.0022. Note that the ∆*actA* strain did not form infectious foci and circularity measurement reflected the shape of infected cells, which at the last time points, rupture and release bacteria into the cell culture medium.

Previous studies that measured the size of *L. monocytogenes* infectious foci by plaque assays and that analyzed infected cells post-fixation by fluorescence or electron microscopy demonstrated the important role of LLO and PLCs in *L. monocytogenes* intercellular spread (30, 31). To establish how LLO and PLCs affect the dynamics of infectious focus formation in real time and on a cell population level, we performed live imaging of HeLa cells expressing a fluorescent plasma membrane marker (Lck-mTurquoise2) infected by fluorescent (RFP) WT *L. monocytogenes* or isogenic *actA*, *hly*, *plcAB*, or *plcABhly* deletion mutants. In these strains, RFP is expressed under the control of the promoter P*actA*, which is regulated by the transcriptional activator PrfA (32). PrfA is allosterically activated by glutathione once bacteria have access to the host cell cytosol, generating bacterial fluorescence (33). Monolayers of cells were infected and imaged 2.0–11.5 h post-infection. We measured the size and circularity of the infectious foci as well as bacterial growth within foci over time (Fig. 1; Movie S1; Fig. S1). WT *L. monocytogenes* focus surface areas were characterized by their near-exponential growth, with the brightest regions localizing at the center of the foci and their anisotropic spread along the periphery due to pioneer bacteria, as previously described (34). As a negative control, we used an ActA-deficient strain, which infects host cells but is unable to undergo intercellular spread. As expected, ActA-deficient *L. monocytogenes* displays linear growth within initially infected cells until they rupture, releasing bacteria into the extracellular space, but there was no formation of bacterial foci. Bacterial growth area was confined within initially infected cells and adopted a more circular shape. As expected, the triple deletion mutant strain (Δ*plcABhly*) failed to infect cells due to its complete inability to escape from the primary internalization vacuole in the absence of PLCs and LLO (Fig. S1B) (35, 36). On the contrary, the LLO- and PLC-deficient strains were each able to escape from primary internalization vacuoles and to spread from cell to cell, although the size and intracellular growth of their infectious foci were markedly decreased compared to the WT strain (Fig. 1; Movie S1; Fig. S1). Interestingly, infectious foci of the LLO-deficient strain still presented anisotropic expansion as observed with the WT strain, whereas foci of the PLC-deficient strain displayed a modest but statistically significant decrease in anisotropic expansion (Fig. 1). Noticeably, PLC-deficient *L. monocytogenes* grew in compacted structures near the periphery of the foci, which was not observed for the other studied bacterial strains (Fig. 1; Movie S1). These spherical configurations suggested that PLC-deficient bacteria grew contained in subcellular vacuolar compartments.

Together, these data show that PLCs and LLO are required for efficient *L. monocytogenes* intercellular spread. In the absence of LLO, the PLCs (and reciprocally, in the absence of the PLCs, LLO) can promote *L. monocytogenes* cell-to-cell spread but with dramatically lower efficiency compared to WT. Furthermore, the distinct phenotypic appearances of foci support that LLO and PLCs influence intercellular spread in different ways. We asked next how LLO and PLCs influence the spatiotemporal dynamics of the donor and recipient cell plasma membranes during single cell-to-cell spread events.

### Simultaneous visualization of donor and recipient host cell plasma membranes reveals two *L. monocytogenes* cell-to-cell spread pathways

**Fig 2:**
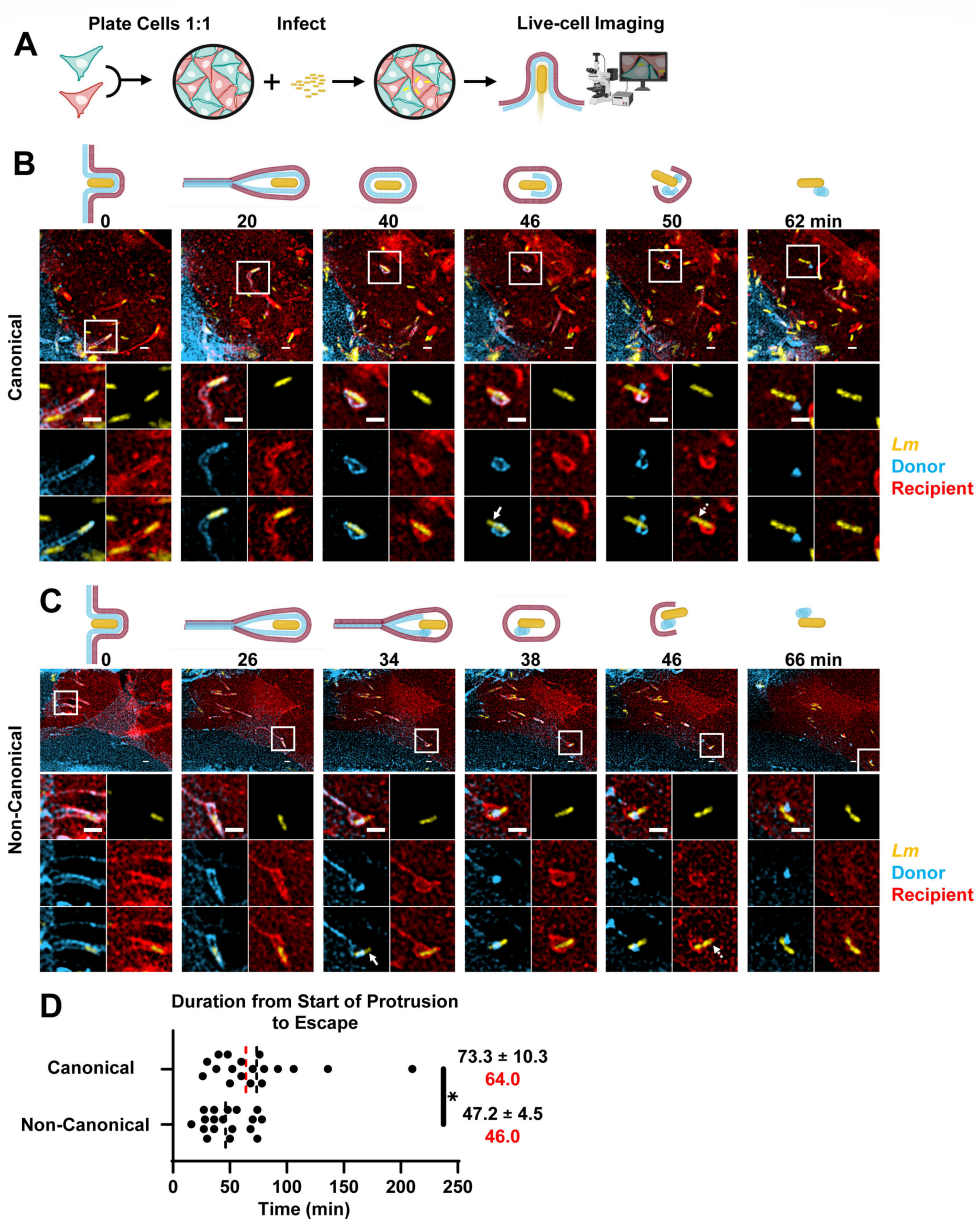
Canonical and non-canonical *L. monocytogenes* cell-to-cell spread pathways. (**A**) Experimental approach to visualize donor and recipient membranes during *L. monocytogenes* cell-to-cell spread by live-cell fluorescence microscopy. (**B and C**) Representative micrographs were selected from movies (Movies S2 and S3) depicting cell-to-cell spread events that occur through the canonical (**B**) and the non-canonical (**C**) pathways. *z*-stack fluorescence images were acquired and deconvolved using the Richardson-Lucy method. For each time point, the pictures correspond to one *z*-plane intersecting the selected *L. monocytogenes*. The start of the protrusion occurred at time 0. Magnified images show *L. monocytogenes* (yellow), donor membrane (blue), and recipient membrane (red), as single fluorochromes or overlays. Solid arrows indicate donor membrane disruption; dashed arrows indicate recipient membrane disruption. Scale bars are 2 µm. (**D**) Duration of canonical and non-canonical pathways measured from the start of protrusion formation to bacterial cytosolic escape. Black dotted lines correspond to the mean ± standard error of the mean, red dotted lines correspond to the median values. Data are from *n* = 36 cell-to-cell spread events across *N* = 4 independent experiments and were analyzed by a linear mixed-effects model. *P = 0.0273.

**Fig 3:**
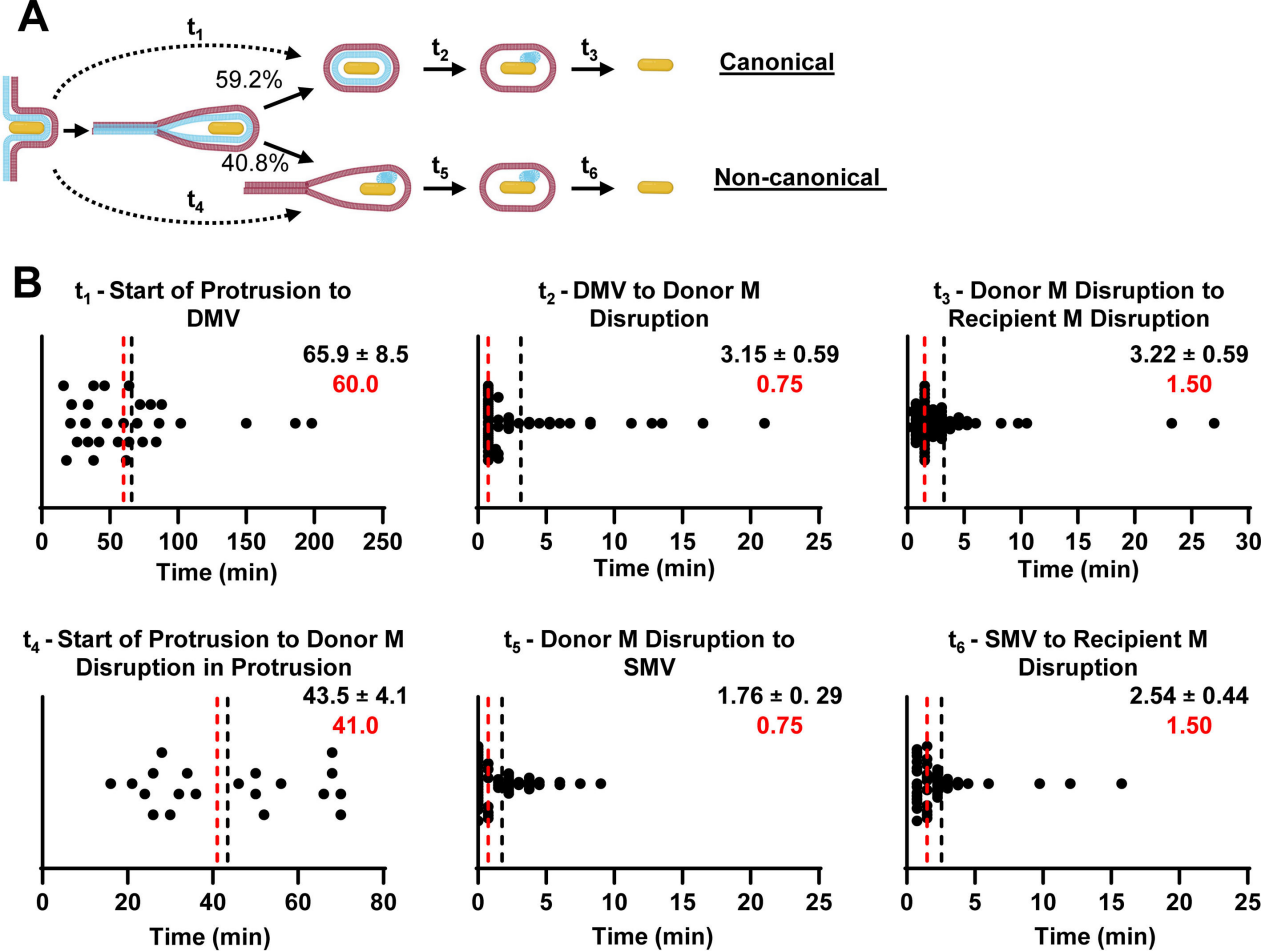
Duration of the cell-to-cell spread stages in the canonical and non-canonical pathways of WT *L. monocytogenes*. (**A**) Representation of the different stages of protrusion dynamics in canonical and non-canonical pathways. (**B**) Quantification of the duration of distinct stages of cell-to-cell spread indicated in panel A. DMV, double-membrane vacuole; M, membrane; SMV, single-membrane vacuole. *t*_1_ and *t*_4_ were measured from long movies, while *t*_2_, *t*_3_, *t*_5_, and *t*_6_ were measured from shorter movies. Black dotted lines and values correspond to the mean ± standard error of the mean; median values are in red. Data are from *n* = 493 cell-to-cell spread events across *N* = 4 independent experiments.

To monitor host cell plasma membrane dynamics within *L. monocytogenes* protrusions, we developed a method in which we could simultaneously visualize and distinguish between the donor and recipient host cell plasma membranes (referred to as donor or recipient membranes). Co-cultured HeLa cells expressing distinct plasma membrane-anchored fluorescent proteins (mTurquoise2, mScarlet-I, or mVenus) were infected with fluorescent WT *L. monocytogenes* (Fig. 2A). We completed this approach in two formats: short movies with sets of images acquired every 45 s for 1 h, to allow monitoring of rapidly occurring events, and longer movies with sets of images acquired every 2 or 3 min for 7 h, to evaluate events with a longer time range. At each time point, we acquired *z*-stacks of three fluorescence images: *L. monocytogenes*, donor membrane, and recipient membrane. After image deconvolution, individual cell-to-cell spread events that took place between cells expressing distinct plasma membrane markers were analyzed to track the different stages of protrusion dynamics: (i) double-membrane protrusion initiation (start), (ii) resolution of the protrusion into a vacuole which coincides with the loss of the protrusion tail, and (iii) disruption of donor and/or recipient membranes until bacteria reach the cytosol (escape). We identified two *L. monocytogenes* cell-to-cell spread pathways (Fig. 2; Movies S2 and S3). We describe the first pathway as “canonical” since it is in agreement with the previously proposed model that successively involves (i) formation and elongation of the double-membrane protrusion; (ii) resolution of the protrusion into DMV concurrent with loss of protrusion tail; and (iii) disruption of the donor membrane and, finally, disruption of the recipient membrane (Fig. 2B) (2, 30). About 59% of intercellular spread events follow the canonical pathway with an average duration of ~73 min. The second pathway, depicted as “non-canonical,” is significantly faster (~47 min) and was observed for 41% of protrusions. The non-canonical pathway is characterized by the following steps: (i) protrusion formation and elongation and donor membrane rupture and collapse while the protrusion tail is still present, (ii) protrusion resolution into a single (recipient)-membrane vacuole concurrent with loss of protrusion tail, and (iii) disruption of the recipient membrane (Fig. 2C). Notably, in both pathways, disruption of the donor membrane happens in a characteristic fashion where it first undergoes a break and then collapses into a globular structure. The kinetics of individual phenotypic stages for each pathway are presented in Fig. 3. For both canonical and non-canonical pathways, the length of time spent in protrusion was the longest stage of intercellular spread (~66 and ~45 min, respectively), whereas after vacuole formation, membrane disruptions occurred in a more rapid fashion (~6.4 and ~2.5 min). These data provide the first report of the dynamics of host plasma membranes during *L. monocytogenes* cell-to-cell spread, confirming the canonical pathway and revealing the existence of the more efficient non-canonical pathway.

### LLO is required for the non-canonical pathway and controls DMV formation as well as donor and recipient cell membrane disruption in the canonical pathway

**Fig 4:**
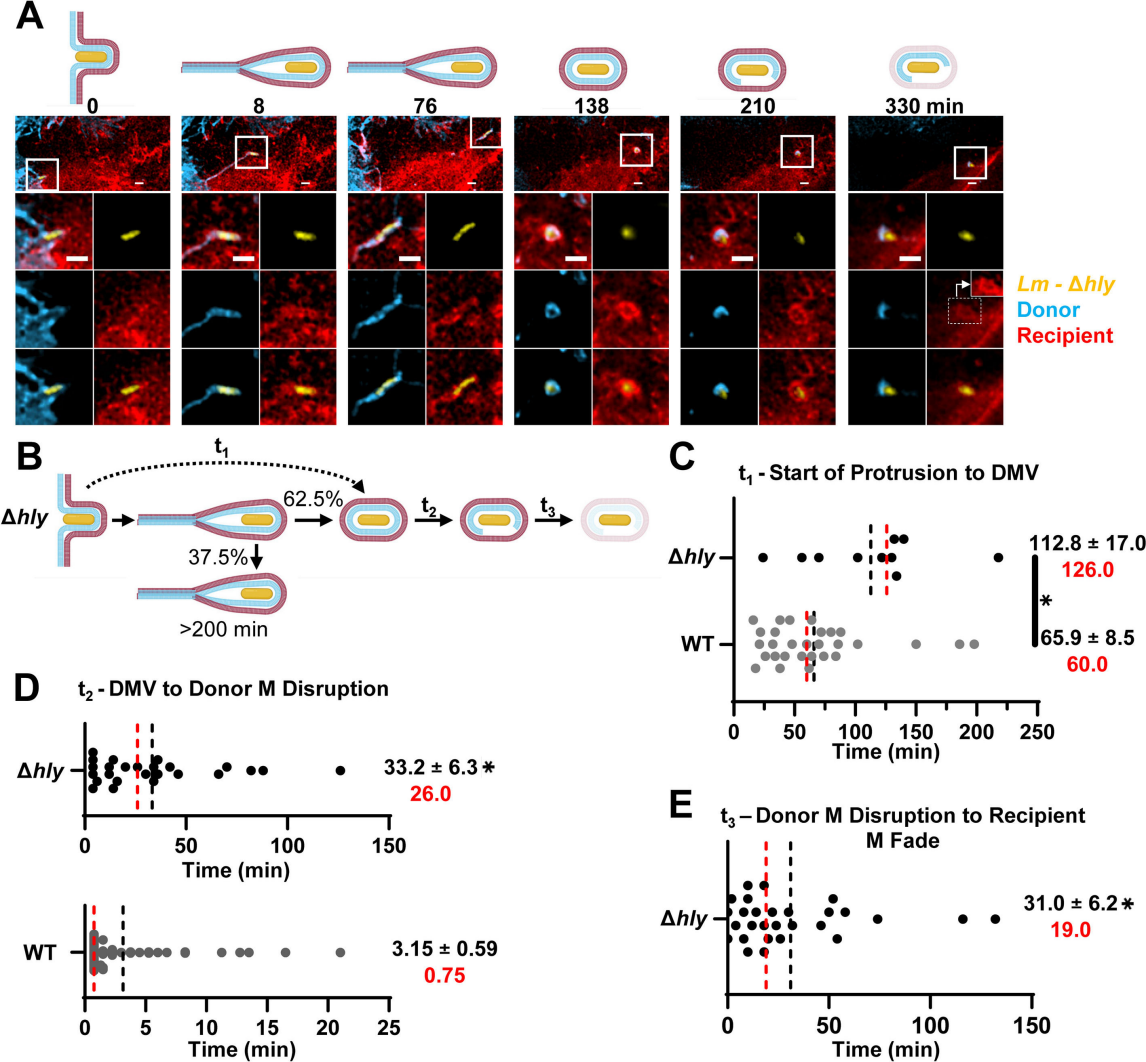
Role of LLO in *L. monocytogenes* cell-to-cell spread pathways. (**A**) Micrographs were selected from a representative movie depicting a cell-to-cell spread event of Δ*hly L. monocytogenes*. *z*-stack images were deconvolved using the Richardson-Lucy method. Magnified images show *L. monocytogenes* (yellow), donor membrane (blue), and recipient membrane (red) as single fluorochromes or overlays. At 330 min, the boxed area shows artificially enhanced fluorescence intensity of the recipient membrane. Scale bars are 2 µm. (**B**) Different stages of protrusion dynamics during Δ*hly L. monocytogenes* cell-to-cell spread. (C–E) Quantification of the duration (min) of distinct stages of cell-to-cell spread events indicated in panel B. DMV, double-membrane vacuole; M, membrane. (**C**) *t*_1_ was measured from long movies. (**D**) *t*_2_ was measured from long movies for Δ*hly* and from short movies for WT *L. monocytogenes*. (**E**) *t*_3_ was measured from long movies. Black dotted lines and values correspond to the mean ± standard error of the mean, red lines and values correspond to the medians. Δ*hly* data are from *n* = 61 cell-to-cell spread events across *N* = 4 independent experiments. Data were analyzed by a linear mixed-effects model comparing duration (min) of cell-to-cell spread events of Δ*hly* to WT *Lm*. *P < 0.05. *t*_1_ *P* = 0.0109, *t*_2_ *P* < 0.0001, *t*_3_ *P* < 0.0001). Of note, *t*_3_ corresponds to the duration from donor membrane disruption to recipient membrane fading of Δ*hly and* therefore is an underestimation of the delayed cytosolic escape of the Δ*hly* strain.

To dissect the role of LLO in cell-to-cell spread, we next analyzed the LLO-deficient *L. monocytogenes* mutant strain (Fig. 4; Movie S4). Due to the extended lifetime of intercellular protrusions formed by the LLO-deficient strain, 1 h-long movies were too short; therefore, we only analyzed 7 h-long movies to fully assess the spatiotemporal dynamics of cell-to-cell spread events. Only the canonical pathway was observed for LLO-deficient bacteria (Fig. 4A and B), and DMV formation was significantly delayed with an average time of ~113 min from protrusion start to DMV (an underestimation due to 37.5% of protrusions that formed for extensive times [>200 min] without observable resolution into a vacuole) in comparison to WT *L. monocytogenes* (~66 min) (Fig. 4C). For the protrusions containing LLO-deficient bacteria that resolved into DMV, there was a major delay in disruption of the donor membrane compared to WT bacteria (~33 min versus ~3 min) (Fig. 4D). Unlike what we reported for the disruption of the donor membrane by WT *L. monocytogenes* (Fig. 2), in the absence of LLO, a single break occurred in the donor membrane, and there was no collapse of the donor membrane. Subsequently, the recipient membrane did not undergo a visible disruption, but instead, the fluorescence persisted for an average time of ~31 min, during which the fluorescence gradually became undetectable (fluorescence fading) (Fig. 4E). In conclusion, our data show that LLO expression is strictly required for the non-canonical cell-to-cell spread pathway. Importantly, in the canonical pathway, LLO exerts significant control on protrusion resolution into DMV and on disruption of both donor and recipient membranes for efficient bacterial escape. Indeed, the total duration of the canonical pathway for the WT strain was ~73 min on average, while for LLO-deficient bacteria, this duration was strictly superior to 210 min.

### PLCs are required for donor membrane disruption and autophagy evasion in canonical and non-canonical pathways

**Fig 5:**
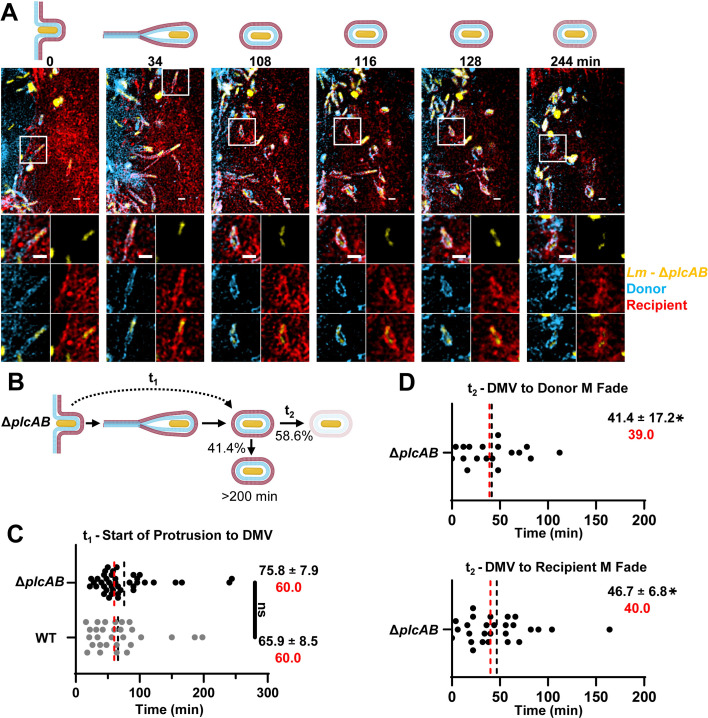
Role of the PLCs in *L. monocytogenes* cell-to-cell spread pathways. (**A**) Micrographs were selected from a representative movie depicting a cell-to-cell spread event of Δ*plcAB L. monocytogenes*. *z*-stack images were deconvolved using the Richardson-Lucy method. The start of the protrusion is indicated as time 0. Magnified images show *L. monocytogenes* (yellow), donor membrane (blue), and recipient membrane (red), as single fluorochromes or overlays. Scale bars are 2 µm. (**B**) Representation of the different stages of protrusion dynamics during Δ*plcAB L. monocytogenes* cell-to-cell spread. (**C and D**) Duration (min) of distinct stages of cell-to-cell spread events indicated in panel B. DMV, double-membrane vacuole; M, membrane. All measurements are from long movies. Black dotted lines and values correspond to the mean ± standard error of the mean; red dotted lines and values correspond to the medians. Δ*plcAB* data are from *n* = 95 cell-to-cell spread events across *N* = 4 independent experiments. Data were analyzed by a linear mixed-effects model comparing duration (min) of cell-to-cell spread events of Δ*plcAB* to WT *L. monocytogenes.* *P < 0.05. ns, not significant. *t*_1_ *P* = 0.4119, *t*_2_ top panel *P* < 0.0001, *t*_2_ bottom panel *P* < 0.0001. Of note, *t*_2_ corresponds to the duration from DMV to donor (or recipient) membrane fading of Δ*plcAB* and therefore is an underestimation of the delayed cytosolic escape of the Δ*plcAB* strain.

**Fig 6:**
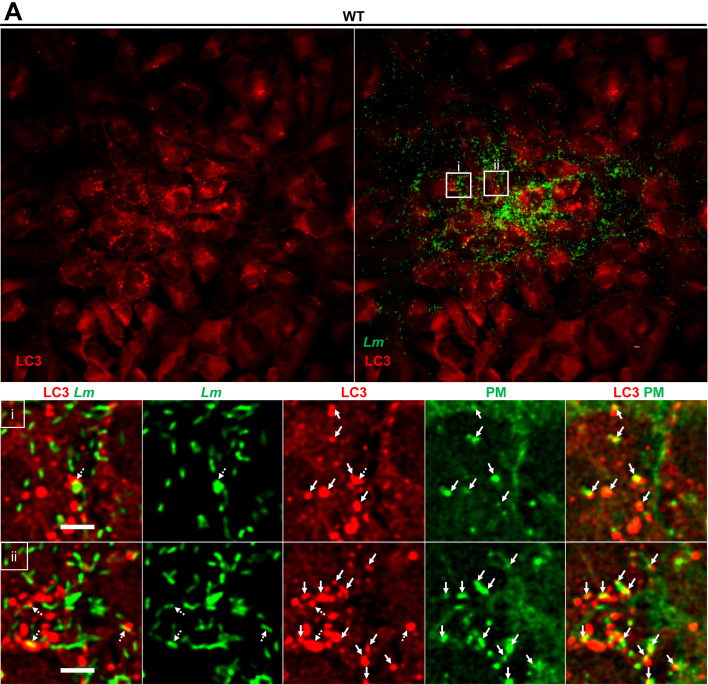
LC3 is associated with cell-to-cell spread membrane protrusion remnants of WT *L. monocytogenes*. HeLa cells expressing plasma membrane marker were infected with WT RFP-*L. monocytogenes*. Cells were fixed, permeabilized, and labeled for LC3. (**A**) Micrographs of *L. monocytogenes* (green) and LC3 (red) at one *z*-plane. (Ai and ii) *z*-stack images were deconvolved using the Richardson-Lucy method. Magnified images show LC3 (red), *L. monocytogenes* (green), and plasma membrane (green) as single fluorochromes or overlays. Solid arrows indicate areas of co-localization of plasma membrane and LC3. Dashed arrows indicate areas of co-localization of *L. monocytogenes* and LC3.

**Fig 7:**
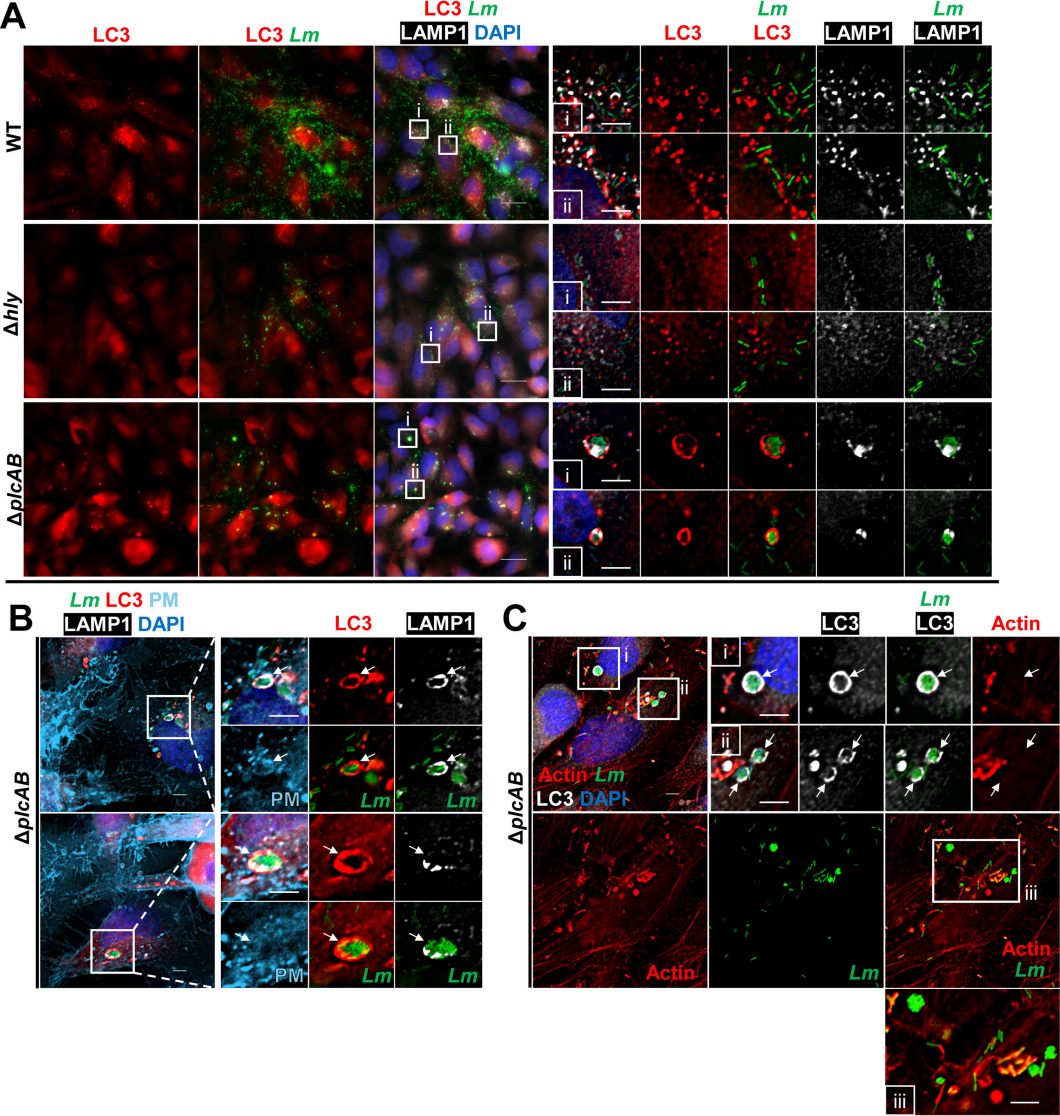
Entrapment of ∆*plcAB L. monocytogenes* in LAMP1-positive autophagosome during cell-to-cell spread. HeLa cells were infected with RFP-WT, -∆*hly*, or -∆*plcAB L. monocytogenes* for 7–10 h. Cells were fixed, permeabilized, and labeled using primary antibodies against LC3 and LAMP1 and secondary fluorescent antibodies, and with 4′,6-diamidino-2-phenylindole (DAPI). (**A**) Micrographs of *L. monocytogenes* (green), LC3 (red), LAMP1 (white), and nuclei (DAPI, dark blue) as single fluorochromes or overlays. Scale bars are 25 µm. (Ai and ii) *z*-stack images were deconvolved using the Richardson-Lucy method. Magnified images correspond to boxed regions from panel **A**. Scale bars in magnified images are 5 µm. (**B**) Micrographs of *L. monocytogenes* (green), LC3 (red), LAMP1 (white), HeLa cell plasma membrane (light blue), and nuclei (dark blue) as single fluorochromes or overlays. Arrows indicate LC3 and LAMP-1 associated vacuoles containing multiple Δ*plcAB L. monocytogenes*. Scale bars are 5 µm. (**C**) Micrographs of *L. monocytogenes* (green), actin (red), LAMP1 (white), and nuclei (dark blue) as single fluorochromes or overlays. (Ci–iii) Boxed areas magnified. Arrows indicate LC3-associated vacuoles containing multiple Δ*plcAB L. monocytogenes*. Scale bars are 5 µm. Data are representative of *N* = 2 independent experiments with two to three internal replicates per experimental condition.

To establish the role of the bacterial phospholipases C in cell-to-cell spread, we monitored cell-to-cell spread dynamics of the isogenic PLC-deficient *L. monocytogenes* strain (Fig. 5; Movie S5). As for the LLO-deficient strain, we analyzed long movies and only observed the canonical pathway (Fig. 5A and B). We found that protrusions of PLC-deficient bacteria resolved in DMV with an average time of ~76 min, which was not statistically different from WT bacteria (Fig. 5C). Remarkably, unlike what we observed for the WT or LLO-deficient strains, there was no noticeable disruption of donor or recipient cell membranes. The DMV either remained fluorescent for extended times with no apparent disruption (>200 min, 41.4% of DMV), or the fluorescence of donor and recipient membranes gradually faded at similar rates after an average time of ~41 and 47 min, respectively (for 58.6% of DMV) (Fig. 5A through D). We also observed confined growth of PLC-deficient bacteria in compacted spherical arrangements comparable to what we previously observed at the focus periphery (Fig. 1A), supporting confined bacterial growth within vacuoles until they rupture, releasing bacteria in the host cell cytosol (Movie S6). To identify the nature of this suspected vacuoles, we next performed LC3 and LAMP1 labeling 7–10 h post-infection by WT and mutant strains. In cells infected by WT bacteria, LC3 puncta were present within infectious focus areas, but not in surrounding uninfected cells (Fig. 6). Interestingly, LC3 puncta very rarely associated with bacteria and frequently co-localized with cell-to-cell spread membrane remnants (Fig. 6). In cells infected by LLO-deficient bacteria, we observed fewer LC3 puncta, but similar to WT, LLO-deficient bacteria were not labeled with LC3 (Fig. 7A). In PLC-deficient bacteria, LC3 puncta were observed in a higher number and larger size within all infectious foci, compared to WT bacteria (Fig. 7A). A large proportion of the PLC-deficient bacteria were entrapped in LC3- and LAMP1-positive vacuoles of different sizes, from enclosing single bacteria to large groups of bacteria (Fig. 7A through C; Fig. S2). LC3- and LAMP1-positive bacteria were not labeled with F-actin, further supporting their lack of access to the cytosol (Fig. 7C). This finding supports the growth of the PLC-deficient mutant in an LC3- and LAMP1-positive non-degradative autophagy compartment from which they ultimately exit to continue their intracellular life cycle but with a significantly lower efficiency than the wild type strain. In summary, PLC expression is strictly required for the non-canonical cell-to-cell spread pathway, as observed for LLO. Unlike LLO, in the canonical pathway, PLCs do not play a significant role in protrusion resolution. These data also demonstrate that PLCs are required for disruption of the donor membrane and in prevention of bacterial entrapment within an autophagosome during cell-to-cell spread.

## DISCUSSION

**Fig 8:**
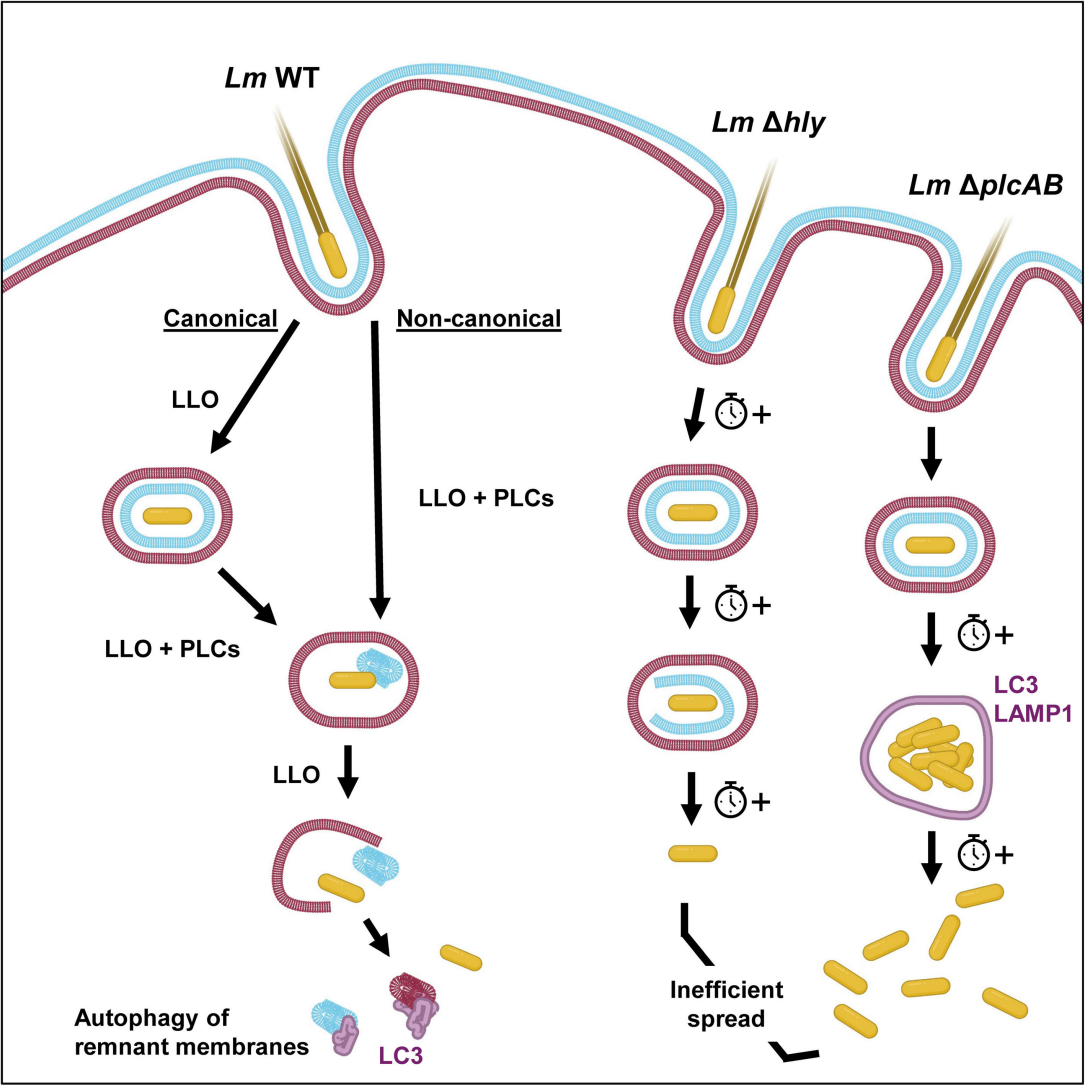
Advanced *L. monocytogenes* cell-to-cell spread model. In agreement with the previous *L. monocytogenes* cell-to-cell spread model (30), the “canonical pathway” successively involves protrusion formation and elongation, protrusion resolution into a double-membrane vacuole, and the successive disruption of donor and recipient membranes. About 60% of protrusions follow the canonical pathway. The second significantly faster “non-canonical” pathway observed for 40% of protrusions is novel and characterized by protrusion resolution into a single-membrane vacuole. In both pathways, the time-limiting stage is from protrusion formation to resolution (~66 min for canonical and ~45 min for non-canonical), but once the vacuole is formed, membrane disruption is relatively rapid (~6.4 min canonical and ~2.5 min non-canonical), which is followed by the entrapment of membrane remnants by the autophagy machinery. In the absence of LLO or PLCs, *L. monocytogenes* cell-to-cell spread is inefficient as previously known. The advanced model proposes that cooperation between LLO and PLCs is required for canonical and non-canonical pathways to mediate donor membrane disruption. Furthermore, LLO controls protrusion resolution and recipient membrane disruption, while PLCs prevent bacterial entrapment into an autophagy compartment. Indeed, protrusion resolution of LLO-deficient (Δ*hly*) *L. monocytogenes* into a DMV and disruption of the donor and recipient membranes are drastically and significantly delayed. Protrusions of PLC-deficient (Δ*plcAB*) *L. monocytogenes* resolve into a DMV that becomes targeted by the autophagy machinery, leading to bacterial growth into a non-degradative LC3- and LAMP1-positive vacuole that eventually ruptures allowing for bacterial cytosolic escape.

*L. monocytogenes* cell-to-cell spread is critical for pathogenesis. Prior studies proposed that the *L. monocytogenes* intercellular protrusion resolves into a DMV, with the donor cell membrane disrupted by PLCs, whereas LLO disrupts the recipient cell membrane (27, 30). This model was deduced from electron microscopy studies, but the relative dynamics of donor and recipient membranes remodeling within protrusions and vacuoles remained undetermined. The present study directly assessed *L. monocytogenes* cell-to-cell spread by simultaneous visualization of donor and recipient membranes in living cells. Our data provide an advanced model for *L. monocytogenes* cell-to-cell spread in human HeLa epithelial monolayers and establish the roles of PLCs and LLO in protrusion resolution, membrane disruption, and escape from the autophagy machinery (Fig. 8).

Live-cell imaging of lipids within membranes is challenging due to their rapid diffusion and constant trafficking. Here we used fluorescent proteins fused to short peptide sequences, which are the site for post-translational lipidation (N-myristoylation and S-palmitoylation), ensuring their stable anchorage via lipid groups to the inner leaflet of the plasma membrane (37). Previous work on *Shigella flexneri* cell-to-cell spread successfully used a similar plasma membrane marker to track intercellular protrusions, but the same fluorescent membrane marker was used in donor and recipient cells (38). The half-life of membrane-bound myristoylated and palmitoylated proteins is several hours (39, 40), making these fluorescent chimeras excellent tools to follow membrane dynamics within protrusions. By co-culturing cells expressing distinct fluorescent proteins, we successfully tracked in real time the relative dynamics of donor and recipient membranes of WT *L. monocytogenes*. A limitation of this approach is that these dynamics can be monitored as long as the lipidated protein remains anchored to the membrane (a few hours). This characteristic limited the time during which long-lived protrusions and vacuoles of the LLO- and PLC-deficient *L. monocytogenes* could be observed, leading to gradual fading of the fluorescence signal of DMV that persisted for several hours. In addition, prolonged 3D imaging of several fluorochromes at short time intervals was accompanied by some photobleaching despite the very low and gentle illumination conditions used in this work. Although we cannot rule out that expression of the fluorescent lipidated proteins does not affect protrusion dynamics, our findings support that this is not the case as the obtained data nicely correlate with previous studies that did not involve fluorescent membrane markers, as detailed below.

Using live-cell 3D imaging, we identified two pathways used by *L. monocytogenes* to escape from the double-membrane protrusion. First, the “canonical pathway,” by which 59% of protrusions resolve into a DMV within ~73 min (Fig. 2), corresponds to the currently proposed *L. monocytogenes* cell-to-cell spread model (27, 30). A new finding in this pathway is that LLO controls DMV closure and donor cell membrane disruption (Fig. 4 and 8). We also confirmed that PLCs are required for disruption of the donor cell membrane and showed that they prevent capture of the cell-to-cell spread vacuole by the autophagy machinery (Fig. 5 to 8), as previously suggested by electron microscopy studies (27, 30). The non-canonical cell-to-cell spread pathway that we identified was followed by 41% of protrusions with an average duration of ~47 min. This pathway is characterized by premature donor cell membrane disruption via dual action of LLO and PLCs, bypassing the requirement for protrusion resolution into a DMV and allowing for an expedited bacterial escape (Fig. 2 and 8). A possible explanation for the non-canonical pathway would be higher secretion levels of LLO and PLCs by a subset of bacteria. The non-canonical pathway could not be identified in previous studies that only analyzed fixed cells and did not distinguish donor from recipient membranes. Thus, these previous studies could not determine if SMV resulted from DMV after donor membrane disruption or from protrusion resolution into a SMV.

Protrusions of the PLC-deficient strain resolved into DMV within a similar time frame to that of the WT strain, suggesting that PLCs play a negligible role in DMV formation. However, the finding that LLO plays a significant role in the resolution of the protrusion into a DMV in epithelial cells was unexpected. DMVs are unlikely to form spontaneously but rather result from active endocytic uptake of the protrusion by the recipient cell. In this line of thought, it was proposed that dynamin- and caveolin-dependent endocytic uptake by the recipient cell is responsible for DMV formation (24, 25). Together, these findings suggest that LLO controls caveolin-dependent endocytic uptake of the protrusion by recipient epithelial cells. Such a conclusion is consistent with the role of LLO in the uptake of the *L. monocytogenes* protrusion by efferocytosis in macrophages (41). In the latter model, membrane perforation by LLO leads to exposure of phosphatidylserine on the outer leaflet of the donor membrane, which is then recognized as an “eat-me signal” by receptor TIM-4 on the recipient macrophage membrane (41). Although HeLa cells are not known to express TIM-4 or to undergo efferocytosis, it is likely that LLO is altering the properties of the donor membrane facilitating the activation of an endocytic pathway in the recipient epithelial cell. It was shown that ActA processing by the *L. monocytogenes* metalloprotease Mpl is important for protrusion resolution into vacuoles, which was independent of PC-PLC (42). It will be important for future studies to establish how ActA and LLO, together, control protrusion resolution.

Another important new finding emerging from our study is that, in addition to PLCs, LLO is important for disruption of the donor cell membrane in both canonical and non-canonical pathways. Again, this could not be previously reported due to the technical limitations of past experimental approaches. Furthermore, we observed that during the spreading of WT *L. monocytogenes*, the donor membrane is disrupted in two stages: (i) an initial break followed by (ii) a rapid and full collapse into a small globular structure. However, in the LLO-deficient strain, there was a break in the donor membrane without full collapse, resulting in the membrane remaining in a “crescent moon” shape. In the absence of PLCs but in the presence of LLO, no donor membrane break or collapse was detected. These observations are in accordance with previous reports of an LLO/PLCs cooperation for the disruption of the primary phagocytic vacuole (28). Also, studies using artificial liposomes incubated with recombinant virulence factors showed that LLO/PC-PLC cooperation leads to a more aggressive and full membrane collapse (43). Therefore, we could show in the more complex environment of a living cell that concerted action of LLO and PLCs is required for efficient donor membrane disruption.

Previous work established that the role of LLO in *L. monocytogenes* escape from the phagosome (primary vacuole) varies between cells (12, 31). Indeed, in the absence of LLO, *L. monocytogenes* can escape from the primary vacuole of some human epithelial cells, including HeLa cells (31), although at much lower efficiency than wild-type bacteria. However, in human and murine macrophages, LLO is strictly required for escape from the primary vacuole (15, 44). Similarly, the mechanisms controlled by LLO during intercellular spreading and the extent to which LLO controls these mechanisms may vary between cell lines and/or cell types. Therefore, it will be important to assess the role of LLO as well as the prevalence of canonical and non-canonical cell-to-cell spread pathways in various cell models.

We were puzzled by the observation that PLC-deficient *L. monocytogenes* grew in groups at the edge of foci, that then suddenly dispersed (Fig. 1; Fig. S1; Movies S1 and S6), strongly supporting their growth in a vacuole. Indeed, the fluorescent membrane marker was detected on some of these bacterial groups (Fig. 7B), but most of the bacterial groups had lost the membrane marker after prolonged time periods. Immunofluorescence labeling further supported that PLC-deficient *L. monocytogenes* were indeed trapped into a LC3- and LAMP1-positive, non-degradative compartment (Fig. 7; Fig. S2). Ultimately, rupture of these compartments may rely on mechanical forces generated by bacterial overcrowding and/or by accumulated production of LLO. Such vacuolar replicative niche is reminiscent of the previously described *Listeria*-containing vacuoles (LisCVs) in epithelial cells and epithelial spacious *Listeria*-containing phagosomes; the latter were characterized by long-term residence of replicating *L. monocytogenes* within LAMP1- and LC3-positive vacuoles (45–47). The LC3 puncta were present in WT and LLO- and PLC-deficient *L. monocytogenes* infectious foci but not in cells outside of the foci. Importantly, higher numbers and larger puncta were observed in foci of the PLC-deficient strain despite the significantly lower bacterial burden. Unlike what we observed with PLC-deficient bacteria, LC3 structures were not associated with WT or LLO-deficient bacteria but frequently co-localized with the fluorescent plasma membrane marker (Fig. 6). Together, these data support that PLC expression is required for *L. monocytogenes* avoidance of autophagy during cell-to-cell spread, in accordance with prior studies showing that PLCs prevent activation of autophagy on *L. monocytogenes* primary phagosomes and prevent entrapment of the cell-to-cell spread vacuole in multilayered membranes in macrophages (27, 48). It is likely that PLCs prevent autophagy activation on the *L. monocytogenes*-containing vacuole*,* but following vacuolar escape, remnants of protrusion membranes are discarded by autophagy in recipient cells (Fig. 6 and 8). This study focused on the combined impact of *L. monocytogenes* PI-PLC and PC-PLC due to their predicted overlapping functions. It will be of interest to determine the role of each bacterial phospholipase in the dynamics of donor and recipient membranes in future studies.

Overall, the present study identifies an approach for studying how intracellular pathogens remodel host plasma membranes during intercellular infection, providing valuable mechanistic insight into real-time dynamics of host-pathogen interactions. We applied this approach to *L. monocytogenes* intercellular spread, leading to an advanced cell-to-cell spread model. This study lays the foundation for future investigations of mechanisms used by diverse intracellular pathogens to remodel host plasma membrane structures during intercellular spread. A deeper understanding of these mechanisms will provide insights into both fundamental cell biological processes and potential therapeutic approaches for controlling infections.

## MATERIALS AND METHODS

### Mammalian cell culture and generation of stable cell lines

To generate single-colony stable HeLa cell (American Type Culture Collection #CCL-2) lines expressing monomeric fluorescent proteins (mTurquoise2, mVenus, or mScarlet-I) anchored to the inner leaflet of the plasma membrane through myristoylation and palmitoylation lipid modification sequence (Lck), the Lck-fluorescent protein (FP) coding sequences were amplified from their respective vectors and cloned into BamHI/EcoRI sites of the pLVX-M-puro vector using the primers BamHI-Lck.Fwd and Lck.Rev described in Table 1. Resulting lentiviral plasmids (pLVX-Lck-FP-puro) were validated by Sanger sequencing (Genomics Shared Resource, The Ohio State University [OSU] Comprehensive Cancer Center). Lenti-X 293T cells were transfected with psPAX2, pMD.2, and pLVX-Lck-FP-puro plasmids to produce lentiviruses. HeLa cells were transduced with lentiviruses and selected for integration through 1 µg/mL puromycin selection. Polyclonal cell lines from wells with at least 70% cell death were isolated, and single colony cell lines were obtained. These cell lines had growth rates comparable to parent HeLa cells (data not shown).

**TABLE 1:** Materials^*a*^

Strain, reagent, or resource	Reference or source	Identifier or catalog number
*L. monocytogenes* strains		
WT 10403S	(49)	10403S
WT (10403S) P*hyper*-YFP	This study	SL71
WT (10403S) P*actA*-RFP	(32)	
WT (10403S) P*actA*-RFP-2	This study	SL105
WT (10403S) P*hyper*-RFP	This study	SL114
Δ*hly*	(50)	DP-L2161
Δ*hly* (DP-L2161) P*hyper*-YFP	This study	SL102
Δ*hly* (DP-L2161) P*actA*-RFP	This study	SL107
Δ*hly* (DP-L2161) P*hyper-*RFP	This study	SL116
Δ*plcAB*	(51)	DP-L6578
Δ*plcAB* (DP-L6578) P*hyper*-YFP	This study	SL113
Δ*plcAB* (DP-L6578) P*actA*-RFP	This study	SL106
Δ*plcAB* (DP-L6578) P*hyper*-RFP	This study	SL115
Δ*plcABhly*	(36)	DP-L2319
Δ*plcABhly* (DP-L2319) P*actA*-RFP	This study	SL108
Δ*actA*	(50)	DP-L3078
Δ*actA* (DP-L3078) P*actA*-RFP	This study	SL111
Primers		
BamHI-Lck.Fwd	This study	5′-taagcaggatccggtgggaggtctatataagcag-3′
Lck.Rev	This study	5′-ctacaaatgtggtatggctg-3′
P*actA*-RFP.Fwd	This study	5′-tcgtttgttgaactaatgggtgc-3′
P*actA*-RFP.Rev	This study	5′-aggcagttattggtgccctt-3′
EagI-*Lm*RFP.Fwd	This study	5′-taagcacggccggctaattaagaagataattaactgctaatcc-3′
Restriction enzymes		
BamHI	New England Biolabs	R0136S
EcoRI-HF	New England Biolabs	R3101S
ApaLI	New England Biolabs	R0507S
KpnI-HF	New England Biolabs	R3142S
EagI-HF	New England Biolabs	R3505S
Plasmids		
Lck-mTurquoise2	Addgene (52)	98822
Lck-mVenus-C1	Addgene, (53)	84337
Lck-mScarlet-I	Addgene (52)	98821
pLVX-M-puro	Addgene (54)	125839
pLVX-Lck-mTurquoise2-puro	This study	
pLVX-Lck-mVenus-puro	This study	
pLVX-Lck-mScarlet-I-puro	This study	
pMD2.G	Addgene (Trono, unpublished)	12259
psPAX2	Addgene (Trono, unpublished)	12260
pPL2	(55)	
pPL2-P*hyper-*YFP	(56)	
pPL2-P*actA*-RFP	This study	
Stable cell lines		
HeLa	ATCC, authenticated by STR profiling, STRA7778	CCL-2
Lck-mTurquoise2 HeLa	This study	
Lck-mVenus HeLa	This study	
Lck-mScarlet-I HeLa	This study	
Lenti-X 293T	Takara	632180
Antibodies/staining		
Anti-LC3	Medical and Biological Laboratories	PM036
Anti-LAMP1	Developmental Studies Hybridoma Bank	H4A3
Alexa Fluor 647-conjugated secondary	Invitrogen	A21235
Alexa Fluor 647-conjugated secondary	Invitrogen	A21245
Alexa Fluor 750-conjugated secondary	Invitrogen	A21037
Alexa Fluor 488 Phalloidin	Invitrogen	A12379
Alexa Fluor 647 Phalloidin	Invitrogen	A22287
DAPI	Sigma-Aldrich	32670
Reagents		
DMEM, high glucose, pyruvate	Gibco	11995065
DMEM, high glucose, HEPES, no phenol red	Gibco	21063029
MEM non-essential amino acids solution	Gibco	11140050
Heat-inactivated fetal bovine serum	Fisher	501527079
Penicillin/streptomycin	Fisher	15140122
Chloramphenicol	Sigma-Aldrich	C0378
Gentamicin	Sigma-Aldrich	G1397
Brain heart infusion broth	Fisher	B11059
Dulbecco’s phosphate-buffered saline	Gibco	14190136
Trypsin EDTA	Fisher	25200072
HEPES	Fisher	15630080
Penicillin G	Sigma-Aldrich	13752
Sodium pyruvate	Gibco	11360070
Ibidi imaging 24-well plate	Ibidi	82426
Thermo Fisher 96-well imaging plate	Fisher	165305
Puromycin	Fisher	A11138
PfuUltra II Fusion High-fidelity DNA polymerase	Agilent	600672
T4 DNA ligase	New England Biolabs	M0202S
Paraformaldehyde solution	Thermo Scientific Chemicals	J19943.K2
Triton-X 100	Thermo Scientific	28314
Software		
Nikon NIS Elements HC/AR	https://www.microscope.healthcare.nikon.com/products/software/nis-elements	
GraphPad Prism 10	https://www.graphpad.com/	
Biorender	https://www.biorender.com/	
Bioinformatics tools		
Trimmomatic	(57)	GitHub: https://github.com/timflutre/trimmomatic
SPAdes	(58)	GitHub: https://github.com/ablab/spades
QUAST		SourceForge: https://quast.sourceforge.net/
Bowtie2	(59)	SourceForge: https://bowtie-bio.sourceforge.net/bowtie2/index.shtml
Samtools	(60)	GitHub: https://github.com/samtools/samtools
HTSlib	(61)	GitHub: https://github.com/samtools/htslib, https://www.htslib.org/
Bcftools	(60)	GitHub: https://github.com/samtools/bcftools

^*a*^ DAPI, 4′,6-diamidino-2-phenylindole; DMEM, Dulbecco's modified Eagle medium.

### *L. monocytogenes* sequencing

WT (10403S) and mutant isogenic Δ*hly* (DP-L2161), Δ*actA* (DP-L3078), Δ*plcAB* (DP-L6578), and Δ*plcABhly* (DP-L2319) *L. monocytogenes* strains were gifts from Dr. Daniel Portnoy (UC Berkeley, CA, USA). The P*actA*-RFP (in 10403S background) *L. monocytogenes* strain was a gift from Dr. Anna Barkardjiev (32). Strains were sequenced to verify their phenotype and genome stability after decades of storage. Twenty nanograms of extracted DNA (Promega Maxwell RSC Cultured Cells DNA Kit, Madison, WI) was used to generate libraries using the Illumina DNA Prep (San Diego, CA) kit and protocol with the following modifications: (i) Illumina’s (M) beads were substituted with (L) beads to obtain larger insert sizes; and (ii) library purification was performed using a 1:1 sample to bead ratio. Samples were barcoded using IDT for Illumina UD Indexes. Metagenomic libraries were sequenced as paired-end, 150 bp run, targeting a minimum of 2 million reads per sample using an Illumina NextSeq2000. The Illumina paired-end reads were trimmed for quality and adapters with Trimmomatic (version 0.36) using the following parameters: “ILLUMINACLIP:adapters.fa:2:30:10 LEADING:3 TRAILING:3 SLIDINGWINDOW:4:15 MINLEN:50.” For genome assembly, the quality-trimmed reads from each sample were assembled with SPAdes (version v3.13.0), with the parameters “--only-assembler –careful.” QUAST (version 5.0.2) with default parameters was used to assess assembly quality. Draft genomes were assessed for proper isogenic deletions, and the sequence of pPL2-P*actA*-RFP was confirmed at the pPL2 integration site (tRNA gene) in the WT P*actA*-RFP strain (55). Single-nucleotide polymorphisms (SNPs) against the parent strain WT *L. monocytogenes* 10403S (NC_017544.1) were determined through alignment of the quality filtered reads to the NC_017544.1 genome using Bowtie2 (version 2.4.1) with default parameters. The resulting SAM file was converted into a sorted BAM file using samtools (version 1.16.1). Next, variant calling was performed with bcftools (version 1.16) by running mpileup with the parameters “-Q 30 -q 42 a FORMAT/AD,FORMAT/DP,INFO/AD” and then running call with the parameters “-m --ploidy 1 -Ov.” The resulting VCF file was compressed using bgzip (htslib version 1.11) and indexed with bcftools. Finally, bcftools view was used to apply a filter and generate the output with SNPs and indels, with these parameters “-v snps,indels -e 'FORMAT/DP < 10'.” DNA extraction, sequencing, and analysis were performed by the Infectious Diseases Institute-Genomics and Microbiology Solutions Laboratory, OSU.

### Generation of *L. monocytogenes* fluorescent strains

We generated WT, Δ*hly*, Δ*plcAB*, Δ*actA*, and Δ*plcABhly L. monocytogenes* strains expressing mRFP or mYFP under the control of P*actA* (P*actA-*RFP) (62) or P*hyper* (P*hyper-*YFP and P*hyper*-RFP) (63). To develop the pPL2-P*actA*-RFP plasmid, the P*actA*-RFP sequence from the WT P*actA*-RFP genome was cloned into KpnI and ApaLI sites of the pPL2 vector using the primers P*actA*-RFP.Fwd and P*actA*-RFP.Rev indicated in Table 1. To develop the pPL2-P*hyper*-RFP plasmid, the mTagRFP codon-optimized sequence was amplified from pPL2-P*actA*-RFP using EagI-*Lm*RFP.Fwd and P*actA*-RFP.Rev primers (Table 1) and cloned into the Eagl and ApaLI sites of pPL2-P*hyper*-YFP plasmid (kindly provided by Dr. Cossart laboratory, Pasteur Institute) (56), thus replacing the YFP sequence with mTagRFP. All developed plasmids were verified by whole plasmid sequencing by Plasmidsaurus using Oxford Nanopore Technology. Plasmids were electroporated into *L. monocytogenes* as previously described (64). Due to missense mutations found in the original WT P*actA*-RFP *L. monocytogenes* strain, a new strain was generated using our developed pPL2-P*actA*-RFP and 10403S WT strain (WT P*actA*-RFP-2). Transformed *L. monocytogenes* colonies were selected on brain heart infusion (BHI) agar plates containing 8 μg/mL chloramphenicol.

### *L. monocytogenes* infections

Overnight cultures of *L. monocytogenes* were diluted and grown to mid-log phase at 37°C in BHI broth. Bacteria were washed three times in Dulbecco’s phosphate-buffered saline (DPBS) and added to mammalian cells in antibiotic- and heat-inactivated fetal bovine serum (HIFBS)-free complete culture medium at the indicated multiplicity of infection (MOI 0.2–10.0). Infected cell cultures were immediately centrifuged at 200 × *g* for 5 min and incubated at 37°C for 1 h to allow for *L. monocytogenes* internalization. Cell cultures were then washed three times with antibiotic-free medium and then cultured with 15 µg/mL gentamicin to kill extracellular bacteria.

### Characterization of bacterial foci

Lck-mTurquoise2 HeLa cells were seeded at 1.3 × 10^4^ in a 96-well Thermo Fisher imaging plate and incubated overnight. Cells were infected with P*actA*-RFP *L. monocytogenes* strains (MOI 0.2–1.0) for 1 h, then washed and incubated in DMEM without phenol red containing 10% HIFBS and 15 µg/mL gentamicin. Cells were placed on the atmosphere-controlled microscope stage (5% CO_2_, 37°C), and images were recorded starting at 2 h post-infection. For each well, 25 fields of view were acquired (mTurquoise2 and mTagRFP fluorescence) every 30 min for 9.5 h using a ×20 objective. Images were stitched together to create a 3.75 × 3.75 mm image of each well. To measure bacterial growth within foci, a threshold was applied to the RFP images to create a binary image of fluorescent bacteria (Bi*Lm*), then the ∑ of surface area within the binary image was measured at each time point. Size of infectious foci was measured by applying the functions “connect objects” then “circular close” to the binary image of fluorescent bacteria (Bi*Lm*)*,* creating a new binary image (Bi*Lm*2) that delineates the foci, and the corresponding area was measured at each time point. To calculate the circularity of the foci, we applied the function “circularity” to the binary image Bi*Lm*2. For all measurements in Fig. 1; Fig. S1, and Movie S1, the same threshold and criteria were applied to all strains and images. Presented data are the averages and standard error of the mean (SEM) of *N* = 3 independent experiments, in which 7–10 foci were analyzed per strain (WT, Δ*actA*, Δ*hly*, and Δ*plcAB*).

### Analysis of donor and recipient membrane dynamics

HeLa cells expressing fluorescent membrane markers were co-cultured at a 1:1 ratio (Lck-mTurquoise2 with Lck-mVenus, or with Lck-mScarlet) in 24-well ibidi imaging plates to reach ~80% confluence at the time of infection. Cells were infected with fluorescent WT or mutant *L. monocytogenes* (P*hyper*-YFP or P*actA*-RFP) at an MOI of 0.2–10.0. After 4 h of infection, cells were incubated with phenol red-free medium containing 10% HIFBS and gentamicin and were transferred to the atmosphere-controlled microscope stage. Imaging locations were selected in infected areas with adjacent cells expressing distinct plasma membrane markers. Live-cell imaging of bacteria traveling and rotating in a three-dimensional space within donor and recipient host cell plasma membranes required the acquisition of large *z*-stack images. To minimize photobleaching and phototoxicity, which would interfere with biological processes, we optimized imaging for gentle illumination of fluorophores while maintaining the required *z*-step and fluorescent signal required for downstream deconvolution. Three fluorescence images were acquired corresponding to fluorescent bacteria and the two membrane markers, as *z*-stacks (11 images with a step of 0.3  µm) using a ×60 water immersion objective. In short movies, images were captured every 45 s for 1 h (three to four movies per independent experiment), and in long movies, images were captured every 2 or 3 min for 7 h (one movie per independent experiment) for a total of *N* = 4 independent experiments per experimental condition. Image deconvolution was performed using the Richardson-Lucy algorithm. Cell-to-cell spread events were tracked for each fluorochrome, and the time points of protrusion formation, closure, and disruption or disappearance of the plasma membrane markers were obtained for each individual cell-to-cell spread event. To track bacteria traveling and rotating in a three-dimensional space, the entire *z*-stack image set was assessed. To generate figures and supplemental movies that most accurately depict the process of tracking protrusions and the dynamic movement of bacteria, a single *z*-plane was chosen, and optimal lookup table settings were applied to each time point.

### Immunofluorescence labeling of infected cells

HeLa cells were seeded in a 24-well Ibidi or a 96-well Thermo Fisher imaging plate to reach ~80% confluence at the time of infection. Cells were infected at an MOI of 0.2–1.0 with fluorescent WT, ∆*hly*, or ∆*plcAB L. monocytogenes* (P*hyper*-RFP) for 1 h and then washed and incubated in DMEM without phenol red containing 10% HIFBS and 15 µg/mL gentamicin. After 7 h or 10 h infection, cells were fixed with 4% PFA in DPBS for 20 min at room temperature (RT), and permeabilized for 5  min with 0.5% Triton X-100 in DPBS. Blocking was performed at RT for 1 h in PBS 10% HIFBS, and cells were labeled with primary antibodies diluted in DPBS 10% HIFBS at 4°C overnight and then washed and labeled with secondary antibodies in DPBS 10% HIFBS at RT for 1 h. All primary antibodies were non-fluorescent, and secondary antibodies were Alexa Fluor-conjugated. F-actin was labeled with Alexa Fluor-conjugated phalloidin. Fluorescence images were acquired in areas of infection as *z*-stacks of 25 images (step size = 0.3  µm) using a ×60 water immersion objective or as *z*-stacks of 7 images (step size = 1.4  µm) using a ×40 air objective.

### Microscope equipment and image processing

All images were acquired using a Nikon Ti2-E widefield microscope equipped with a temperature, humidity, and CO_2_-controlled blackout enclosure. Ten excitation wavelengths (Spectra III pad 365, 440, 488, 514, 561, 594, 640, and 730  nm) and emission filter sets for 4′,6-diamidino-2-phenylindole (435 ≤ emission ≤ 485 nm), mTurquoise2 (460 ≤ emission ≤ 500 nm), GFP (515 ≤ emission ≤ 555 nm), mVenus/YFP (from 520 ≤ emission ≤ 560 nm), mTagRFP (from 573 ≤ emission ≤ 648 nm), and Alexa Fluor 647 (677 ≤ emission ≤ 711 nm) imaging with a high-speed wheel; back-thin illuminated SCMOS camera (Orca-Fusion BT, Hamamatsu) of resolution of 5.3 megapixels; and nano-positioning Piezo Sample Scanner (Prior). Fluorescence images were acquired in gray scale and were color-coded. NIS Elements High Content Software was used for image acquisition, and NIS Elements Advanced Research Software was used for image analysis.

### Statistical analyses

For statistical analysis of *Lm* focus size and *Lm* growth in Fig. 1, data were first log10 transformed to reduce skewness. For *Lm* focus size, *Lm* growth, and focus circularity, a linear mixed-effect model was used for each individual outcome comparison to take account of the correlation among observations within each experiment measured over time. Data are averages ± SEM from *N* = 3 independent experiments with 7–10 foci analyzed per experimental condition and experiment. Cell-to-cell spread data in Fig. 2 to 5 are the average duration (min) of *N* = 4 independent experiments. In each independent experiment, three to four short movies (WT) and one long movie (WT, Δ*hly*, and Δ*plcAB*) were analyzed. With mutant strains, less cell-to-cell spread events occurred due to decreased bacterial numbers. Furthermore, the long duration of cell-to-cell spread events and fluorescence fading with these mutant strains limited the number of events that could be measured. In total, 493, 95, and 61 bacteria were tracked for WT, Δ*hly*, and Δ*plcAB* strains, respectively. In all figures, each dot represents an individual cell-to-cell spread event. A linear mixed-effects model was used for analysis across all data sets to account for observations from the same replicate. The model was then used to compare duration differences between groups. For all analyses, a *P* value of <0.05 was used as the cutoff for significance. Across all graphs, black dotted lines and values represent the mean ± SEM, and red dotted lines and values represent the median.

## Data Availability

*L. monocytogenes* sequence data have been deposited to the National Center for Biotechnology Information under BioProject ID PRJNA1274948.

## SUPPLEMENTAL MATERIAL

Fig. S1 mbio.01183-25-s0001.tif Analyses of WT and mutant *L. monocytogenes* focus formation.

Fig. S2 mbio.01183-25-s0002.tif Entrapment of ∆*plcAB L. monocytogenes* into LC3-positive autophagosomes.

Supplemental Legends mbio.01183-25-s0003.pdf Legends for supplemental movies and figures.

Movie S1 mbio.01183-25-s0004.mp4 Formation of WT and mutant *L. monocytogenes* infectious foci.

Movie S2 mbio.01183-25-s0005.mp4 Representative movie of WT *L. monocytogenes* canonical pathway presented in Fig. 2B.

Movie S3 mbio.01183-25-s0006.mp4 Representative movie of WT *L. monocytogenes* non-canonical pathway presented in Fig. 2C.

Movie S4 mbio.01183-25-s0007.mp4 Representative movie of ∆*hly L. monocytogenes* cell-to-cell pathway presented in Fig. 4A.

Movie S5 mbio.01183-25-s0008.mp4 Representative movie of the ∆*plcAB L. monocytogenes* cell-to-cell spread presented in Fig. 5A.

Movie S6 mbio.01183-25-s0009.mp4 Compact structures of Δ*plcAB* during cell-to-cell spread burst open.

ASM does not own the copyrights to Supplemental Material that may be linked to, or accessed through, an article. The authors have granted ASM a non-exclusive, world-wide license to publish the Supplemental Material files. Please contact the corresponding author directly for reuse.
