# Supplementary figures and images for: Role of listeriolysin O and phospholipases C in L. monocytogenes intercellular protrusion dynamics, resolution, and autophagy avoidance
Source: mBio. 2025 Aug 18;16(9):e01183-25. doi: 10.1128/mbio.01183-25 (PMC12421892; doi:10.1128/mbio.01183-25)

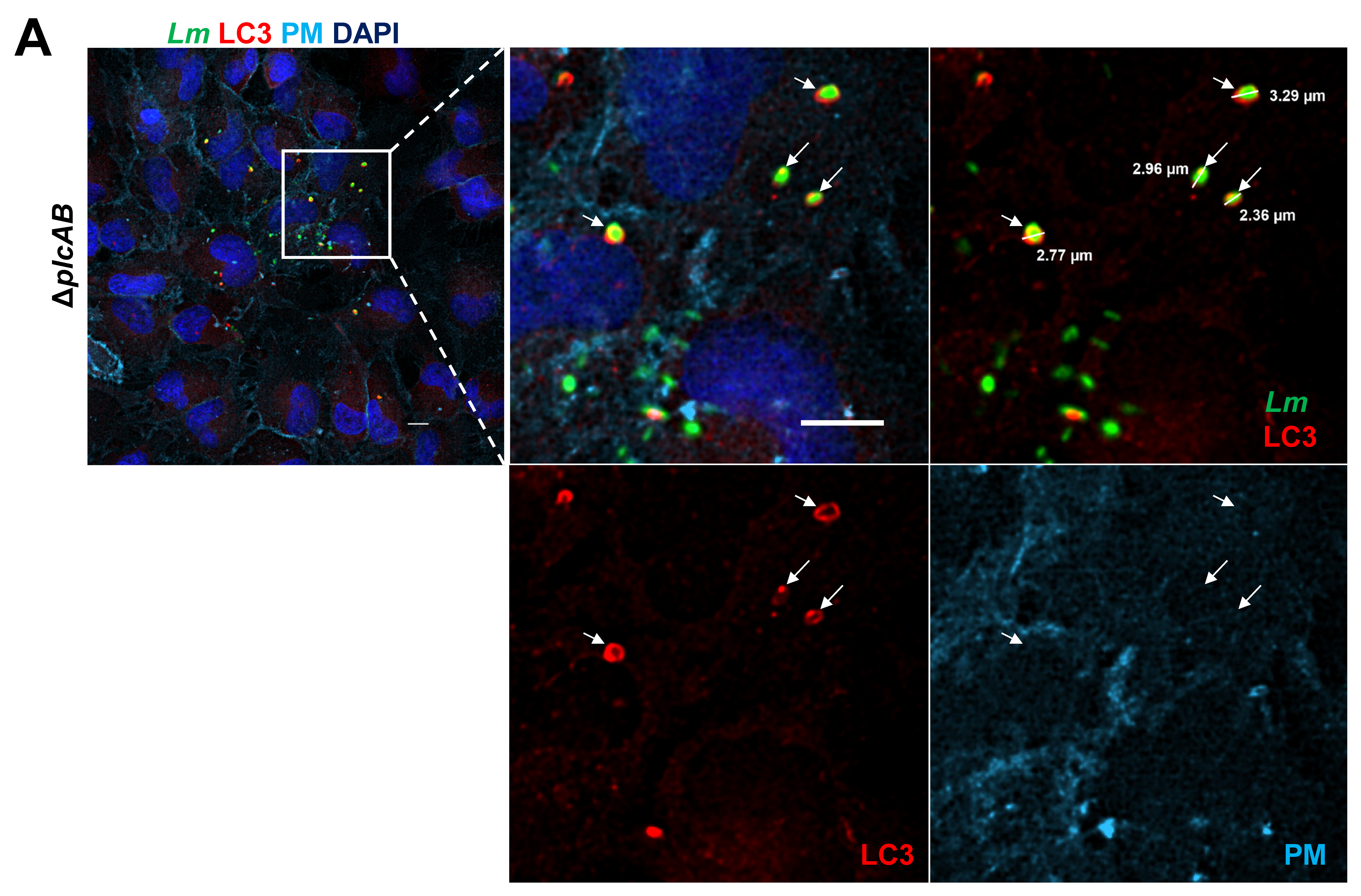

Supplement: Fig. S2 — Entrapment of ∆plcAB L. monocytogenes into LC3-positive autophagosomes. [file mbio.01183-25-s0002.tif]
