# Supplementary material for: Role of listeriolysin O and phospholipases C in L. monocytogenes intercellular protrusion dynamics, resolution, and autophagy avoidance
Source: mBio. 2025 Aug 18;16(9):e01183-25. doi: 10.1128/mbio.01183-25 (PMC12421892; doi:10.1128/mbio.01183-25)
Supplement: Supplemental Legends — Legends for supplemental movies and figures. [file mbio.01183-25-s0003.pdf]

SUPPLEMENTAL FIGURE LEGENDS

Supplemental Movie 1: WT and mutant *L. monocytogenes* infectious foci formation.

Plasma membrane labeled HeLa cells (Lck-mTurquoise2, blue) were infected with WT, Δ*actA*, Δ*hly*, or Δ*plcAB L. monocytogenes* that express RFP upon access to the cytosol (red). Timelapse of foci growth (left) and representative binary that correspond to intracellular *L. monocytogenes* growth (Bi*Lm*, middle) and binary that corresponds to foci size (Bi*Lm*2, right). Scale bars are 100 μm.

Supplemental Figure 1: Analyses of WT and mutant *L. monocytogenes* foci formation.

HeLa cells expressing fluorescent plasma membrane marker (Lck-mTurquoise2, blue) were infected with WT, Δ*actA*, Δ*hly*, Δ*plcAB,* or Δ*plcABhly L. monocytogenes* that express RFP upon access to the cytosol (red). (**A**) Data corresponding to each measured foci are presented on the graphs of foci size, foci circularity, and bacterial growth. Black lines on the graphs are representative of foci micrographs that are shown in Figure 1A. Data are from N=3 independent experiments with 7-10 foci per strain and independent experiment. (**B**) Stitched micrograph (3.75 x 3.75 mm) of cells infected by Δ*plcABhly L. monocytogenes* after 12 h; (**Bi**) Magnified image showing area of Δ*plcABhly L. monocytogenes* infection. Below micrographs are the binary images of *L. monocytogenes* (Bi*Lm, white*). Scale bars are 100 μm.

Supplemental Movie 2: WT *L. monocytogenes* canonical cell-to-cell spread.

Representative movie of WT *L. monocytogenes* canonical pathway presented in Figure 2B. One z-plane intersecting the selected *L. monocytogenes* is shown per time point. Arrow follows the cell-to-cell spread event being tracked. 1 – start of protrusion, 2 – resolution into a DMV, 3 – disruption of the donor membrane with intact recipient membrane, 4 – disruption of the recipient membrane. *L. monocytogenes* (yellow), donor membrane (blue), recipient membrane (red). Scale bar is 2 μm.

Supplemental Movie 3: WT *L. monocytogenes* non-canonical cell-to-cell spread. Representative movie of WT *L. monocytogenes* non-canonical pathway presented in Figure 2C. One z-plane intersecting the selected *L. monocytogenes* is shown per time point. Arrow follows the cell-to-cell spread event being tracked. 1 – start of protrusion, 2 – disruption of the donor membrane with protrusion tail still present, 3 – resolution into a single (recipient) membrane vacuole, 4 - disruption of the recipient membrane. *L. monocytogenes* (yellow), donor membrane (blue), recipient membrane (red). Scale bar is 2 μm.

Supplemental Movie 4: Δ*hly* *L. monocytogenes* cell-to-cell spread. Representative movie of Δ*hly* *L. monocytogenes* cell-to-cell pathway presented in Figure 4A. One z-plane intersecting the selected *L. monocytogenes* is shown per time point. Arrow follows the cell-to-cell spread event being tracked. 1 – start of protrusion, 2 – resolution into a DMV, 3 – disruption of the donor membrane. *L. monocytogenes* (yellow), donor membrane (blue), recipient membrane (red). Scale bar is 2 μm.

Supplemental Movie 5: Δ*plcAB* *L. monocytogenes* cell-to-cell spread. Representative movie of the Δ*plcAB* *L. monocytogenes* cell-to-cell spread presented in Figure 5A. One z-plane intersecting the selected *L. monocytogenes* is shown per time point. Arrow follows the cell-to-cell spread event being tracked. 1 – start of protrusion, 2 – resolution into a DMV. *L. monocytogenes* (yellow), donor membrane (blue), recipient membrane (red). Scale bar is 2 μm.

Supplemental Movie 6: Compact structures of Δ*plcAB* *L. monocytogenes* during cell-to-cell spread burst open. Timelapse of one z-plane showing the disruption of a group of Δ*plcAB* *L. monocytogenes.* Arrows follow the cell-to-cell spread compartment being tracked. *L. monocytogenes* (yellow), plasma membrane (blue and red). Scale bar is 5 μm.

Supplemental Figure 2: Entrapment of Δ*plcAB* *L. monocytogenes* into LC3-positive autophagosomes. HeLa cells expressing fluorescent plasma membrane marker (Lck-

mTurquoise2, light blue) were infected with Δ*plcAB* fluorescent *L. monocytogenes* (RFP, green) for 7 h. Cells were fixed, permeabilized, and labeled for LC3. Z-stack images were acquired and deconvolved using the Richardson-Lucy method. (**A**) Micrograph of *L. monocytogenes* (green), HeLa cell plasma membrane (light blue), LC3 (red) and nuclei (dark blue) as single fluorochromes or overlays at one z plane. Arrows indicate example areas of LC3-positive vacuoles containing *L. monocytogenes*. The measured diameter of the structures (white lines) are provided on LC3 (red) and *L. monocytogenes* (green) overlay image.
